# Genome evolution of Kaposi sarcoma-associated herpesvirus (KSHV)

**DOI:** 10.1128/jvi.01950-24

**Published:** 2025-04-16

**Authors:** Razia Moorad, Alice Peng, Justin Landis, Linda J. Pluta, Patricio Cano, Angelica Juarez, Dirk P. Dittmer

**Affiliations:** 1 Lineberger Comprehensive Cancer Center and Department of Microbiology and Immunology, School of Medicine, University of North Carolina at Chapel Hill 6797, Chapel Hill, North Carolina, USA; University of Toronto, Toronto, Ontario, Canada

**Keywords:** Kaposi's sarcoma, human herpesviruses, KSHV, AlphaFold, PacBio, evolution, human herpesvirus 8, Kaposi sarcoma-associated herpesvirus, Kaposi sarcoma herpesvirus

## Abstract

Kaposi sarcoma (KS) is the most common cancer in people living with HIV (PLWH), particularly in sub-Saharan Africa (SSA), where Kaposi sarcoma herpesvirus (KSHV or human herpesvirus 8 [HHV-8]) is endemic. In KSHV endemic areas, the overall survival of KS patients has changed little over the past 20 years. A phylogenetic analysis of available full-length viral genomes (*n* = 164) identified two different virus lineages that co-circulate in KSHV endemic regions today. Their sequences differ from the GenBank reference sequence and those of common laboratory strains, which originated in the 1990s in the US and Europe. Targeted short-read sequencing accuracy was validated by PacBio-based long-read sequencing to resolve repeats. This analysis identified over 1,000 single nucleotide variants (SNV) in a new 14-member sequence collection from tumor biopsies and blood in Malawi with 127 ± 32 (median ± SD) SNV per genome. Most were private, i.e., specific to one individual’s virus. Within each of the two lineages, KSHV continues to evolve over time and across national borders by genetic drift and recombination. Analyses of shared SNVs by AlphaFold2 predicted some changes in the conformation of key viral proteins. These findings may help our understanding of herpesvirus evolution.

**IMPORTANCE:**

To understand viruses, the field needs to know their genetic makeup. To develop mechanistic models, targeted therapies, and vaccines, we need comprehensive and up-to-date sequence information on the viral strains that circulate where the diseases appear today. Our knowledge of Kaposi sarcoma herpesvirus (KSHV) sequence distribution and evolution is behind that of other human herpesviruses and RNA viruses. Here, we add to community knowledge using new technologies and artificial intelligence.

## INTRODUCTION

Kaposi sarcoma herpesvirus (KSHV), formally known as Kaposi sarcoma-associated herpesvirus, is a double-stranded DNA virus. KSHV is also called human herpesvirus 8 (HHV-8). KSHV is the etiological agent for all forms of Kaposi sarcoma (KS) (1), for KS-associated diseases (KADs) (2, 3), the plasmablastic variant of multicentric Castleman’s disease (MCD), and primary effusion lymphoma (PEL). KSHV also causes acute diseases such as KS-immune reconstitution inflammatory syndrome (KS-IRIS) and KSHV inflammatory cytokine syndrome (KICS) (reviewed in reference 4). There is no preventative vaccine against KSHV and no therapeutic vaccine against KS (reviewed in reference 5). KS is susceptible to broad-spectrum anti-herpesvirus agents such as ganciclovir, foscarnet, and cidofovir; however, these are not currently used to prevent or treat KS. There is no “cure” for KSHV or KS, as herpesviruses establish molecular latency for life.

In sub-Saharan Africa (SSA), where KSHV is endemic and where the human immune deficiency virus (HIV) remains epidemic, KS is seen in children, adults, and the elderly with or without HIV co-infection (6). Here, KSHV is transmitted in infancy; seroprevalence can exceed 80% before sexual debut, and malaria is considered a cofactor for virus acquisition (7–11). KS is one of the most common cancers in the region (12).

With the widespread availability of combination antiretroviral therapy (cART), the 2000s witnessed a decline in the incidence rates of KS in men who have sex with men (MSM) populations (13). Today, however, KS incidence rates have stabilized. KSHV is firmly entrenched and in high-risk groups, seroprevalence approaches 40% (14). KS remains the most common cancer in people living with HIV (PLWH) worldwide. This stabilization of disease incidence, rather than continued decline, is due to several factors. First, KS incidence rates, independent of HIV, have always been age-dependent. Classic KS is a disease of the elderly in areas with KSHV prevalences upwards of 5%, such as in the Levant or 19th century Vienna. Organ-transplant-associated KS emerges independent of HIV (reviewed in reference 4). Lastly, as the initial cohort of PLWH ages, KS risk increases independent of HIV-related immune suppression. While cART alone slows the progression to AIDS and, by restoring CD4 counts, can cause KS regression in one-third of cases, other PLWH experience KS despite HIV suppression and near-normal CD4 counts (15–19). Today, “a high number of Kaposi sarcoma cases and disparities in Kaposi sarcoma outcomes persist in certain populations in the United States (20).” In 2020, KS was the most common cancer in men in Malawi, accounting for 16.7% of all new cancer cases and 5.7% in females, regardless of HIV status; in Uganda, KS incidence rates accounted for 17.1% of all new cancer cases in men and 7% of new cases in women (21).

As KS and KAD continue to determine overall mortality in PLWH, KSHV deserves continued attention, and KSHV sequence information requires continued validation. The question arises: how similar are the KSHV strains circulating in PLWH today to the original type specimen and the tool isolates that drive basic KSHV research, drug and vaccine development? The first step toward answering this question is a reexamination of the original KSHV isolates with third-generation long-read sequencing by synthesis methods.

The first complete KSHV genome, GenBank accession number U75698, was derived from the Epstein-Barr virus (EBV)/KSHV-coinfected BC-1 PEL cell line in 1996 (22, 23): KSHV^BC-1^. The U75698 consensus sequence was obtained by Sanger-sequencing of a cosmid and lambda library. Next, the KSHV genomes from two primary KS lesions were obtained (U93872 and AF148805). These were also generated by Sanger-sequencing of cosmid or lambda libraries. Neither viruses nor cells corresponding to these two KS derived genomes were ever cultured or clonally expanded. The reference KSHV genome is strain KSHV^GK18^, based on GenBank entry AF148805, represents overlapping cosmids cloned from a KS biopsy. The sequence is oriented as the unique (U) region followed by a single copy of the terminal repeat (TR). The mean number of terminal repeats and authentic ends of the linear virion genome were determined later and patched on. As noted in AF148805, “some copies of TR lack 39 bp” and “74 bp (were) deleted in cos19 and cos32, and also in the majority of DNA from the original biopsy; this disrupts the transmembrane anchor domain of the K8.1 protein; shown as wild type (undeleted).” Hence, it is possible that AF148805 was derived from a pool of defective genomes and repaired post hoc based on sequences from PEL cells. AF148805 became the current reference sequence, which is designated NC_009333. No one has proven experimentally that the NC_009333 sequence represents a replication-competent virus.

Two PEL cell lines deserve special attention: BCBL-1 and JSC-1. Both cell lines yield high levels of infectious KSHV virions. BCBL-1 only carries KSHV, and JSC-1 carries KSHV and EBV (24, 25). Replication-competent bacmids have been derived from these cell lines. These are BAC16 (GQ994935, MK143395, and MK733609) from the JSC-1 cell line (26) and BAC36 (HQ404500) and its derivatives from the BCBL-1 cell line (27). These bacmids are single clones of a viral episome and can be stably propagated in *Escherichia coli* (save for the terminal repeat region). The null hypothesis is that while PEL cell lines and KSHV genomes carried in them are under constant selection for faster growth, KSHV genomes carried in bacteria with intact DNA repair and replication genes are only subject to neutral genetic drift.

According to the International Committee on Taxonomy of Viruses (ICTV), the family Herpesviridae belongs to the order Herpesvirales (28). The Herpesviridae includes three subfamilies: Alphaherpesvirinae, Betaherpesvirinae, and Gammaherpesvirinae. KSHV is of the genus Rhadinovirus, species Rhadinovirus humangamma8.

The community currently recognizes two types of Rhadinovirus humangamma8 based on the K15 sequences on the right-end side: the predominant (P) and minor (M) types (29, 30). The two forms of ORF-K15 have only 33% overall amino acid identity to one another but retain their conserved intracellular signaling motifs. The immediately adjacent ORF75 typically segregates with the M and P types of K15. Both K15 and ORF75 are leftward open reading frames (ORFs). A dual polyA site separates the leftward transcripts for K15 and ORF75 from the rightward K14/vGCPR transcripts. The historical designation as “type” is unfortunate since most evolutionary biologists use lineage as the next subdivision. Nevertheless, the designation “type” is used for KSHV and, more famously, human papillomavirus strains.

We had previously published the clinical data associated with a prospective cohort of HIV-positive KS patients in Malawi (31). Here, we describe the KSHV genomes from their tumor biopsies and plasma samples. We reported on the two African lineages of KSHV previously (32). Here, this finding is confirmed and extended in an independent cohort.

We provide findings of structural implications of shared non-synonymous single nucleotide variants (SNV), as modeled by AlphaFold. AlphaFold first performs a genetic database search based on the template sequence and second generates multiple sequence alignments (MSA), with regions of homology weighted by importance (attention). Third, a structured database search generates an MSA of aligned homologs per residue pair. These MSAs are imputed into the neural network block called the Evoformer, producing a Nseq × Nres array (Nseq is the number of sequences; Nres is the number of residues) and a Nres × Nres array, which represents the MSA and residue pairs, respectively (33). The Evoformer derives a structural hypothesis by exchanging information within the MSA and the residue pairs and extracts the spatial and evolutionary relationships of the protein template. The Evoformer then produces updated MSAs and residue pair representations, repeating the process several times before progressing to the structure module. Lastly, an AlphaFold2 structure is built in the structure module, followed by a local refinement to produce the final structure. While we are cautious about interpreting these models, the data predict that some African-lineage SNVs have functional consequences. Others change the virion surface and thus targets for antibody responses.

AlphaFold3 is now publicly available and free to use for any protein sequence. It can be used to explore the implications of structural variation. We explore three proteins as examples of how the new African Lineages of KSHV may alter virus protein structure. How these structural changes translate into functional phenotypes, however, is beyond even the best *in silico* predictions.

1. The ORF49 protein cooperates with ORF50 in activating several KSHV lytic promoters; additionally, ORF49 can activate the JNK/p38 pathways (34, 35). The crystal structure of ORF49 is solved (5IPX.pdb; 2.8 Å) (36), providing a robust basis for AlphaFold2 predictions.
2. The ORF22, or glycoprotein H (gH), is one of eight glycoproteins associated with the viral envelope (37). The other envelope glycoproteins include gB (ORF8), gM (ORF39), gL (ORF47), gN (ORF53), ORF28, ORF68, and K8.1 (22). The gB, gH, and gL play crucial roles in viral attachment, fusion, and entry. These proteins are highly conserved across all herpesviruses, which aided in training AlphaFold. KSHV ORF22/gH has functional, sequence, and structural homologs in EBV, herpes simplex virus 1 (HSV-1), and human cytomegalovirus (HCMV), namely BXLF2, UL22, and UL75, respectively (22, 38). The gH and gL proteins form a non-covalently linked complex. The gL protein is required for the processing and intracellular transport of gH, indicative of a conserved, co-evolving interaction surface. The crystal structure of gH and gL in complex with the ligand binding domain (LBD) of EphA2 has been solved (39). There are three published X-ray structures for EBV BXLF2, two for HSV-1, one for pseudorabies virus, and three for HCMV UL75, which forms part of the pentameric entry complex. Neutralizing antibodies to the pentameric complex prevent HCMV infection (40, 41).
3. The ORF34 protein interacts with several other proteins, including ORF18, ORF23, ORF24, ORF31, and ORF66 (42). The interaction between ORF34, ORF31, and ORF23 is essential for late gene transcription: deletion of ORF34 results in the loss of progeny viruses. These proteins are highly conserved in EBV and HCMV (43) and bind together to form the viral pre-initiation complex (vPIC), which recruits the cellular RNA polymerase II. ORF34 acts as a scaffold for the vPIC proteins. The central region of ORF34 binds cellular transcription-associated factors, whereas the C-terminus of ORF34 recruits ORF24. ORF24 is a TATA box-binding protein homolog that binds to the TATT sequence in the late gene promoters of KSHV (42–44).

How does one assess the quality of the structure prediction? AlphaFold2 provides multiple scoring metrics, such as the predicted local distance difference test (pLDDT), representing the predicted side-chain torsional angles and accuracy per structure residue (33). The scoring ranges from 0 to 100, where regions of a pLDDT >90 are considered highly accurate. Regions with a pLDDT between 70 and 90 provide a good backbone structure, and regions with a pLDDT between 50 and 70 are modeled with low confidence. Regions with a pLDDT below 50 have low confidence and should not be interpreted.

Another confidence score is the predicted aligned error (PAE). This has two meanings. If there exists a structure, also called a ground truth, PAE measures how well a prediction matches the true structure for each amino acid position in three-dimensional space. This changes for each amino acid position within a protein. Since AlphaFold is based on sequence alignments, more and more closely related sequences will have higher PAE scores. Whereas, PAE is a prediction, root mean square deviation (RMSD) is measured between exactly two final structures. If there is no structure to measure the AlphaFold prediction against, PAE is a computational estimate of how confident the model is about the prediction. In Fig. 5C and 6B, the color at position x, y indicates the AlphaFold2 expected position error at residue x if the predicted and actual structure were aligned at residue y. A low PAE for residue pairs x and y from two different domains indicates that AlphaFold2 had confidently predicted their relative position and orientation. Conversely, a high PAE for residue pairs x and y would indicate low confidence in their predicted positions and orientation. Below, both a structural model and error estimators are reported.

## MATERIALS AND METHODS

### Sample collection

Tissue biopsies from KS skin lesions and plasma samples were collected from 137 participants (31). PBMCs were prepared as cell blocks by centrifugation, with the addition of a thrombin solution for cryopreservation and biopsies embedded in formalin-fixed paraffin-embedded (FFPE) blocks.

### DNA isolation

DNA was extracted from FFPE curls using the QIAmp DNA FFPE Tissue Kit (Qiagen, #56404). DNA was extracted from plasma samples using the Roche MagNA Pure Compact Instrument (Roche, #0373114601). Ten microliters of a control plasmid, Fly2.0 (Addgene #117418) (1.0 × 10^5^ copies/μL), was added to each sample to serve as a control for DNA extraction efficiency. The quantity and quality of the DNA and RNA were assessed by Nanodrop (Thermo Fisher Scientific), Qubit (Thermo Fisher Scientific), and TapeStation (Agilent Technologies).

### Short-read sequencing

DNA library preparation and sequencing from plasma samples were performed using the automated approach on the Ion Torrent Genexus Integrated Sequencer (Thermo Fisher Scientific; Pub. No. MAN0017912), using custom primers to enrich for KSHV. For FFPE samples, library preparation and sequencing were automated on the Ion Torrent Ion Chef and S5 Sequencer with default parameters using custom primers (Thermo Fisher Scientific). Barcodes, primers, and adapter sequences are removed before FASTQ sequence data files are output from the Ion Torrent S5 and Genexus sequencers, respectively. Previously reported primers and PCR conditions (32) were used to amplify and Sanger sequence the GC-rich and repetitive regions not captured by targeted short-read sequencing.

### Long-read sequencing

Approximately 2.5 ug of genomic DNA was fragmented using G-Tube (Covaris) following the technical note “Covaris g-Tube DNA Shearing for SMRTBell Prep Kit 3.0.” Fragmented DNA was then purified using 1× SMRTbell cleanup beads. The size was determined by TapeStation. Concentration was determined by Qubit. The Hi-Fi library was made using the SMRTbell Prep kit 3.0 (procedure and checklist 102-166-600 REV02 MAR2023). Sequencing was conducted per the manufacturer’s instructions (SMRTLink user guide—102-278-200 Version 01 [April 2022]) and SMRT sequencers operations guide (101-774-06 Pacific Biosciences of California, Inc. [“PacBio”]).

### Quality trimming, filtering, and read mapping

FASTQ reads were imported into CLC Genomics Workbench v23.0.2 (Qiagen Inc.). If the quality control report indicated a specific number of bases of poor quality, this number was used for the trim function. If the quality control report did not yield a particular, fixed number of bases to trim, reads were trimmed by removing low-quality base pairs (quality limit = 0.05), including 40 nucleotides at the 5′ terminal and 10 nucleotides at the 3′ terminal. All sequences shorter than 50 nucleotides were filtered out. Next, reads were mapped to the KSHV genome (NC_009333) using the CLC Genomics Workbench v23.0.2 (Qiagen) map-to-reference tool with the following parameters: no reference masking and default mapping options (match score = 1, mismatch score = 2, insertion cost = 3, deletion cost = 3, length fraction = 0.5, similarity fraction = 0.8). The mapped reads were trimmed to the reference. Non-specific matches were ignored. Duplicate mapped reads were removed using default parameters on the remove-duplicate-mapped-reads tool on CLC Genomics Workbench v23.0.2. The CLC Extract Consensus Sequence tool was used to create a consensus sequence. The low coverage threshold was set to three (this was adjusted based on the low coverage areas and the number of reads mapped there in the map file). Conflicts were resolved by voting using quality scores. In the GenBank records, low coverage regions are indicated with “N.” For the phylogenetic analysis of proteins and structure prediction, the open reading frames (ORF) in the consensus sequence were repaired, and SNVs resulting in internal stop codons were corrected. The final, repaired, and annotated sequences were used for further analysis and deposited in GenBank (Table 1).

**TABLE 1:** KSHV whole genome sequence metrics^*a*^

GenBank accession no.	KSP	Type	Age	Gender	No. of sequencing reads	No. of reads after trim	No. of mapped reads	Duplicate mapped reads	Avg. read length	Fold coverage
PQ899225	KSP009	Plasma	33	Male	1,538,048	264,000	251,720	179,991	138	457
PV032230	KSP015	Plasma	49	Male	1,344,479	223,940	210,959	59,578	140	214
PV032229	KSP012	Plasma	41	Male	1,323,840	111,788	108,407	46,496	150	118
PV061047	KSP090	Plasma	42	Male	1,213,127	109,667	91,928	35,438	131	87
PV016613	KSP002*	Plasma	49	Male	1,410,476	395,816	373,504	107,524	139	376
PV032228	KSP010	Plasma	37	Male	1,484,612	148,002	122,055	48,903	146	129
PV032227	KSP008	Plasma	35	Male	1,484,539	230,510	212,436	66,490	144	221
PV016612	KSP001	Plasma	42	Female	1,318,410	195,553	186,986	112,975	146	198
PV061044	KSP024	FFPE	37	Male	572,368	175,078	130,953	89,215	136	129
PV061045	KSP022	FFPE	41	Male	940,920	436,769	371,591	266,280	150	403
PV188213	KSP017	FFPE	37	Male	27,660,787	24,309,935	23,809,123	4,021,265	142	24,468
PV016611	KSP021	FFPE	28	Male	23,723,381	20,459,107	18,268,644	15,213,321	140	18,507
PV061046	KSP031	FFPE	53	Male	24,656,689	31,302,400	30,784,848	4,089,032	126	28,078
PQ899226	KSP002*	FFPE	49	Male	3,497,329	2,851,552	2,090,124	1,655,918	139	2,103

^*a*^ The metadata and mapping results of the sequenced KS samples from Malawi. As indicated, samples were isolated from plasma and KS lesions in FFPE blocks. The sequences were submitted to GenBank, and their accession numbers can be accessed as described in Materials and Methods. * indicates two samples from the same person.

### Variant calling

Variant calling was performed using two orthogonal methods. First, using raw reads, variants, including single nucleotide variants (SNVs), multiple nucleotide variants (MNVs), and insertions and deletions (INDELs), were called using the Basic Variant Detection tool on CLC Genomics Workbench v23.0.2 (Qiagen), with default parameters. SNVs with a minimum average Phred quality score >20, a minimum frequency >35%, and a minimum coverage of >40 reads were identified. A base quality filter with a minimum central quality of 20 and a minimum neighborhood quality of 15 was selected. Pyro-error variants were removed in homopolymer regions of a minimum length of three nucleotides. Second, the SNV calls variant calling on the MAFFT alignment of finished consensus sequences was performed using the find variations/SNPs tool implemented in Geneious v11.1.5 (Dotmatics, Inc.).

### Phylogenetic analysis

The phylogenetic tree was constructed using the high-coverage KSHV genomes generated in this study and other previously published KSHV genomes isolated from Africa. These include those isolated from Zambia (45), Uganda (46, 47), Malawi (32), and Cameroon (48). Additional publicly available genomes isolated from the USA and Japan were included in the analysis along with the reference genome NC_009333 (49).

The KSHV sequences used herein were retrieved using “human gammaherpesvirus 8,” which is the official GenBank taxonomy of KSHV (NCBI Taxonomy ID 37296) across all fields AND filtering by sequence length greater than 100,000. The search result changes with calendar time. For batch download by accession number, the NCBI ncbi-entrez-direct command was used. Note that the GenBank naming rules do not follow the naming conventions of the ICTV (https://ictv.global). The ICTV 2022 taxonomy for KSHV is Duplodnaviria, Heunggongvirae, Peploviricota, Herviviricetes, Herpesvirales, Orthoherpesviridae, Gammaherpesvirinae, Rhadinovirus, and Rhadinovirus humangamma8.

All genomes were aligned using MAFFT, implemented in Geneious v9.1.8 with default parameters (50). The conserved long unique region (LUR) from ORF16 to ORF58 was extracted from the multiple alignments of 164 genomes, resulting in 65,840 bp of continuous sequence, used to infer the Bayesian Maximum Likelihood (ML) tree implemented in Beast v1.10.4 (51, 52). The HKY substitution model, with estimated base frequencies, and the gamma site heterogeneity model were selected to estimate site substitution rates. A strict clock and a constant size coalescent restriction were chosen to construct the ML tree. The ML tree was rooted in the KSHV reference genome NC_099333. The phylogenetic tree representing the left and right side of the LUR (total length is 33,299 bp and 49,517 bp, respectively) was extracted from MAFFT whole genome alignment and constructed using RAxML, implemented in Geneious v.9.1.8, with default parameters (53). The ML trees were constructed using the same parameters described above.

The Splits Tree analysis was performed to investigate conflicting phylogenetic signals and was performed on the ML tree alignment of the LUR using SplitsTree4 (54). The neighbor-net split network was constructed using uncorrected P character transformation and excluded gap sites. Statistical analysis included performing 1,000 bootstrap replicates and the Phi test for evidence of recombination.

### AlphaFold analysis

AlphaFold2 (AF2), V2.3.1, was downloaded from the Google DeepMind GIT repository and installed on a high-performance computing server. UCSF ChimeraX v1.6.1 was used to generate models of the same ORF proteins. KSHV ORF proteins with a non-synonymous variant (Table 2) were modeled using protein sequences from a Malawian isolate in the African subtypes A (MZ712180) and B (MZ712182) identified in the phylogenetic tree, including the reference genome NC_099333. Default monomer-PTM parameters were selected with the option to use PDB structures in the alignment and energy minimizations to optimize the structure. The PDB files of the best-ranking protein structure models were viewed using Pymol v2.5.4. Updated predictions can be obtained using the public AlphaFold3 server at https://alphafoldserver.com.

**TABLE 2:** Variants identified in the KSHV LUR^*a*^

Count	Position	Reference	SNV	ORF	Amino acid	Lineage
1	43962	G	T	ORF25	Ala363Ser	A
2	48197	G	C	ORF27	Leu75Phe	A
3	48308	G	T	ORF27	Glu112Asp	A
4	54964	T	A	ORF34	Leu64Gln	A
5	55059	G	A	ORF34	Glu96Lys	A
6	59464	C	T	ORF39	Val271Ile	A
7	61165	T	A	ORF40	Ser253Arg	A
8	61698	A	G	ORF40	Lys431Arg	A
9	70640	C	A	ORF48	Val281Lue	A
10	75091	T	G	K8	Val48Gly	A
11	76356	G	A	K8.1	Val115Met	A
12	78886	T	A	ORF55	Glu221Val	A
13	81515	G	A	ORF56	Ala661Thr	A
14	81531	C	T	ORF56	Ala666Val	A
15	82616	A	G	ORF57	Asn114Asp	A
16	88416	G	A	vIRF-4	Val165Ala	A
17	88431	T	C	vIRF-4	Tyr160Cys	A
18	88438	G	C	vIRF-4	Gln158Glu	A
19	93230	A	T	vIRF-2	Phe293Lue	A
20	93346	C	T	vIRF-2	Val255Met	A
1	38427	G	A	orf22	Asp406Asn	A&B
2	55446	G	T	ORF34	Ala225Ser	A&B
3	63357	C	T	ORF42-43	Gly566Ser	A&B
4	70300	C	A	ORF48	Cys394Phe	A&B
5	70792	C	T	ORF48	Ser230Asn	A&B
6	92640	A	G	vIRF-2	Leu490Ser	A&B
1	30759	G	C	ORF16	Ser173Thr	B
2	52693	T	C	ORF32	Ser397Leu	B
3	54954	T	A	ORF34	Cys61Ser	B
4	92107	T	C	vIRF-2	Ile668Val	B
5	92124	C	G	vIRF-2	Trp662Ser	B

^*a*^ The 31 variants identified in the LUR of the Malawian samples are described in this study. In subtype A, 20 variants were identified, five in subtype B, and six variants were shared between subtypes A and B. Position numbers are based on the reference genome (NC_009333).

## RESULTS

### Validated genomes from the pre-NGS era increase rigor, robustness, and reproducibility

**Fig 1:**
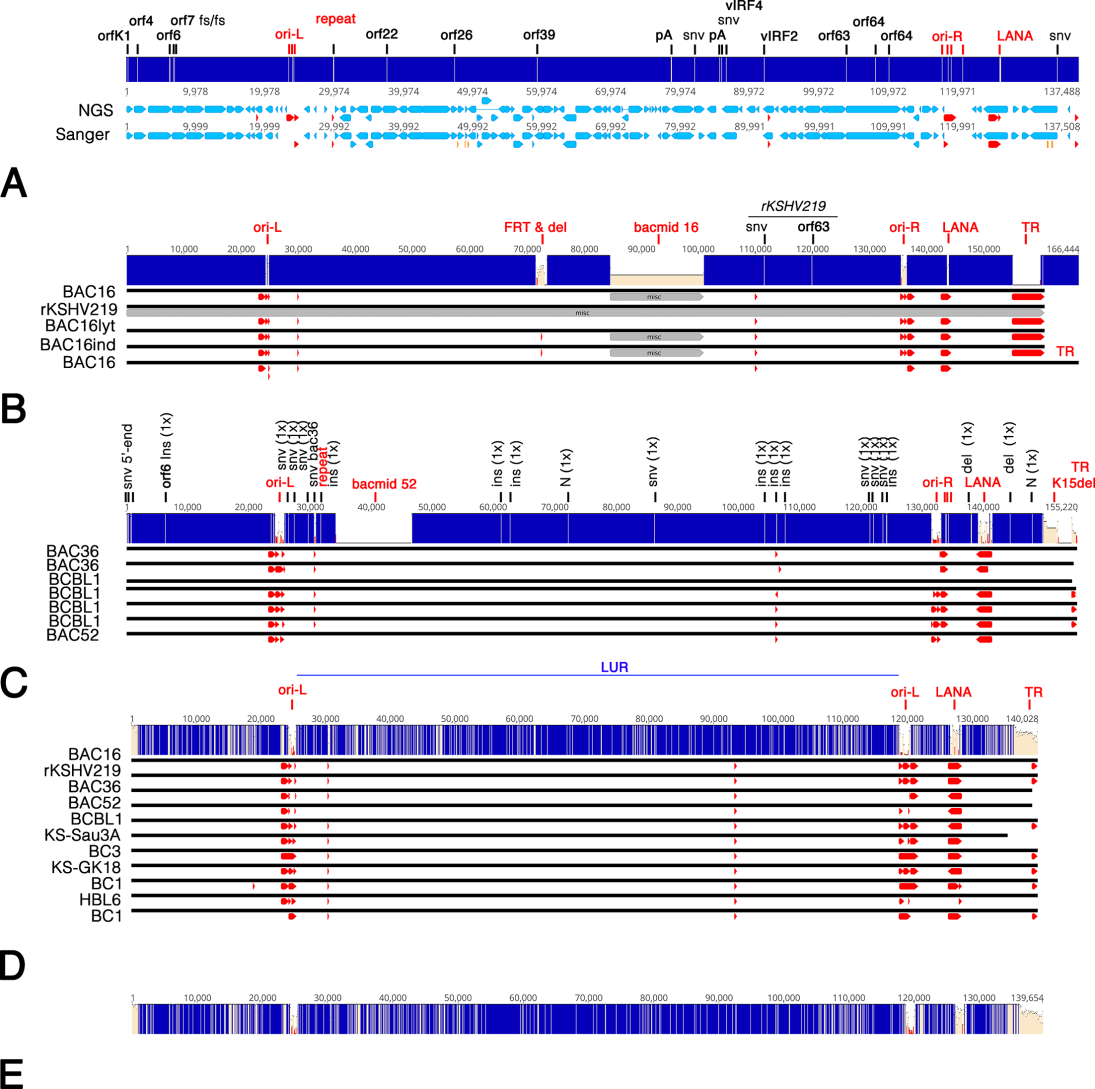
Overview of KSHV sequences. (**A**) Comparison of BC1 isolates U75698 obtained by Sanger-sequencing (Sanger) of a cosmid library and MK7333609 obtained by genome-enriching NGS (NGS). MK7333609 is missing the first 20 nucleotides present in U75698. The ORFs are shown in blue and repeat sequences in red. Individual changes are indicated as white lines in the identity bar and annotated above. Regions of 100% identity are in blue. (**B**) Comparison of non-identical bacmid BAC16 whole genome sequences (GQ99435, KY246443, KY246444, MK208823) and the rK219 isolate (KF588566). Entry OK358814 was 100% identical to GQ994935. Individual changes are indicated in the identity bar and annotated above. (**C**) Comparison of non-identical bacmid BAC36 and BCBL-1 whole genome sequences HQ404500, JX228174, MN205539, MT936340, MZ712172, OR117738, OR333977. (**D**) Comparison of early PEL, KS, and bacmid-contained KSHV genomes: JSC-1^Bac16^ (GQ994935), JSC-1^rKSHV219.1^ (KF588566), BC-1^Russo^ (U75698), BC-1^unc^ (MK733607), BC3 (MK87631), BCBL-1^PacBio^ (OR117738), BCBL-1^Bac36^ (HQ404500), BCBL-1^Bac52^ (OR333977), KS^GK18^ (NC_009333), KS^Sau3a^ (U93872), HBL6 (OR573937). Individual changes are indicated in the identity bar. Also shown is the LUR (from ORF16 to ORF58) between the two Ori-Lyt sites comprising 65,840 bp of continuous sequence. (**E**) Identity graph for just the PEL isolates of (**D**).

Most KSHV whole genome sequences in GenBank were derived by short-read next-generation sequencing (NGS), also called sequencing-by-synthesis. These include multiple, independent entire genome sequences from the same PEL cell lines. Figure 1A compares the KSHV^BC-1^ consensus derived targeted by short-read NGS (MK733607) in 2019 to the Sanger-sequencing derived sequence for KSHV^BC-1^ (NC_003409, U75698) published in 1996 (22). SNVs outside the repeat regions appeared at random. All ORFs stayed “in-frame,” resulting in single-nucleotide and amino acid SNVs. None yield truncated proteins. There were *n* = 50 (0.05%, equivalent to a PHRED value of Q40) nucleotide changes between the original Sanger sequence (U75698) and the short-read NGS, not counting variation in the repeat regions. Whether these are sequence errors in the short-read sequencing, the original GenBank entry, or whether the BC1 episomes accumulated mutations while in continuous culture is unknown.

Only the intended mutations and repeat regions differed among the different BAC16 genomes derived from the PEL cell lines JSC-1 by short-read sequencing (55) (Fig. 1B). Outside the repeat regions, all bacterially-propagated, single-origin viral genomes differed by two SNVs from the rKSHV219.1 maintained in mammalian cells and created in the mammalian JSC-1 cell line “by transfecting JSC-1 cells with pQ219, a plasmid which contains a 4.8 kb segment of KSHV DNA (from BC1 cells) with the RFP/GFP/PURO cassette inserted between ORF K9 and ORF 57 at a site that sequence data indicates does not contain a gene” (56). Based on this alignment, we estimated the minimal error due to neutral genetic drift in bacteria for high-coverage, high-confidence sequences at 2/160,465 or 0.0012%. The bac-propagated viral genomes were 99.9998% identical. This result instills confidence in KSHV bacmid-derived viral mutants and lays to rest concerns that KSHV bacmids are unstable in *E. coli*. There was no evidence of selection against toxic proteins or genome fragments in *E. coli*, except the TR.

Among the different published BAC36 and BCBL-1 genomes, the changes were single SNVs and short INDELs in one or another record but not shared across multiple GenBank entries. These were likely due to sequencing errors. The alignment confirmed the designed mutations, such as a deletion of K15 (57), insertion of the BAC52 backbone (58), and variation in the repeat regions (Fig. 1C). The BAC36 clone has a duplicated unique region fragment inserted in the TR (59). At the time of writing, the following KSHV strains are available as a genome sequence in GenBank and as biological material in public repositories: KSHV^BC-1^, KSHV^BCBL-1^, KSHV^JSC-1^, and KSHV^HBL6^. Of those, the KSHV^BCBL-1^ and KSHV^JSC-1^ were experimentally proven as infectious and replication-competent.

Variation was much more considerable among a representative collection of pre-2000 PEL and KS-derived KSHV genomes (Fig. 1D). The median difference for 55 pairwise comparisons was 1,977 bp, with a mean ± SD of 2,212 ± 1,131 bp. Much of the difference resides in the ends, as different sequence entries include more or less nucleotides at the extreme 5′ end (approximately ±20 bp) and the 3′ end. To account for the possibility that KS isolates introduce extra variation, the multiple alignment was repeated with just the nine PEL isolates (Fig. 1E). Even among the KSHV PEL isolates collected in a similar data range, variation was noticeable. The medium pairwise similarity was 98%, with a mean ± SD of 98% ± 0.88%. Note that 1% of 139,654 bp is still 1,396 individual SNVs. These nucleotide sequences form the historical sequence space and framework in which current experimental work is conducted.

### Additional, full-length viral genomes from PEL cell lines

**Fig 2:**
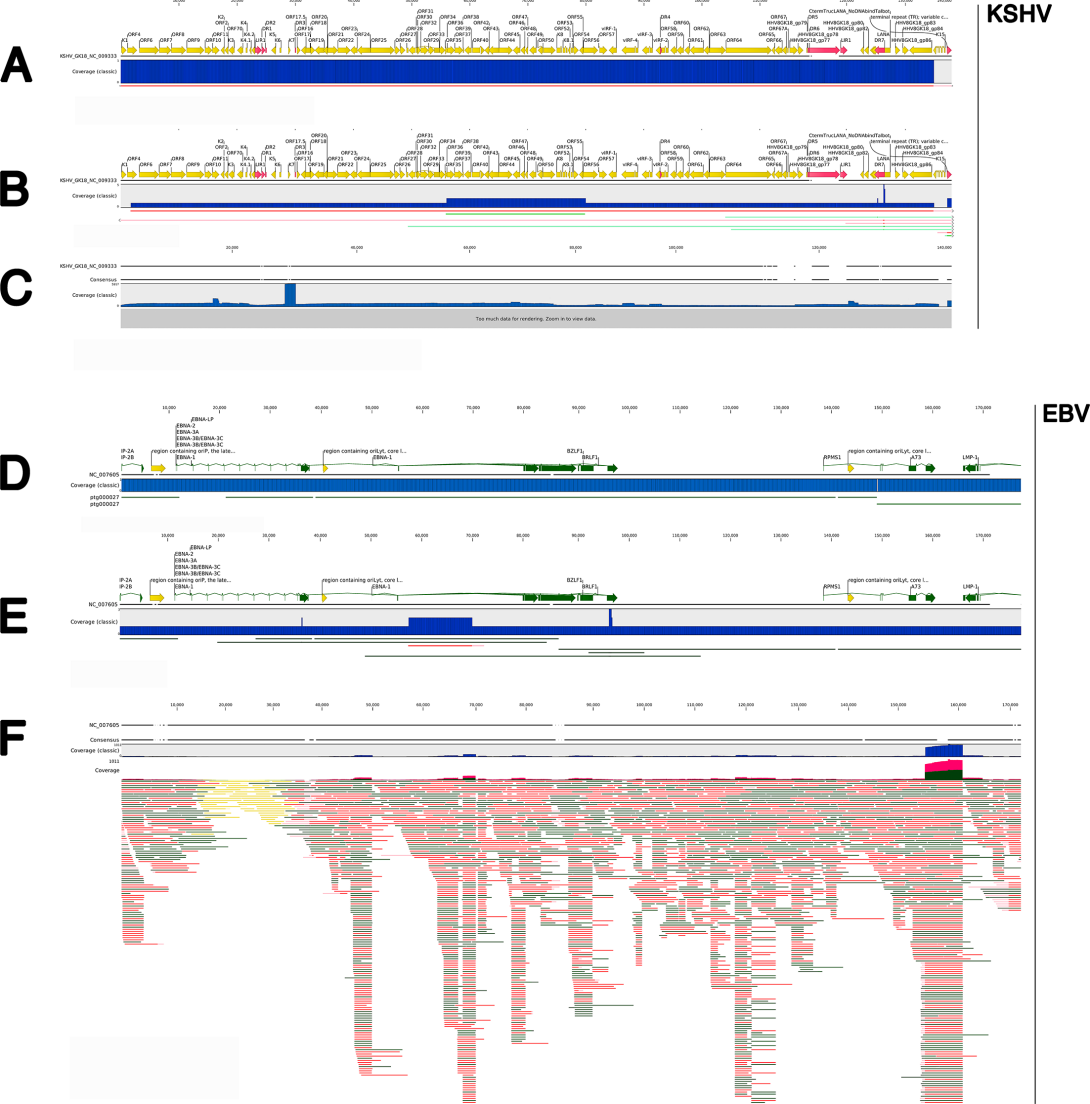
Alignment summary of untargeted, PacBio-derived KSHV and EBV genomes for BC-1 cells. (**A**) _BC1_KSHV^PacBio^ *de novo* assembled from human-depleted reads. The lines diagrammed below indicate *de novo* assembled contigs in either the forward (green) or reverse (red) direction. (**B**) _BC1_KSHV^PacBio^ *de novo* assembled from all reads. The lines diagrammed below indicate *de novo* assembled contigs in either the forward (green) or reverse (red) direction. (**C**) _BC1_KSHV^PacBio^ consensus based on long-read sequencing mapped to GenBank reference NC_009333, a K15 type P strain, vs BC1, which is the M strain. (**D**) _BC1_EBV^PacBio^ *de novo* assembled from human-depleted reads. The ptg00027 designation refers to *de novo* assembled contigs, which are diagrammed as lines below. (**E**) *De novo* assembled from all reads, with the black lines below indicating the longest contigs originating from the assembly. (**F**) Individual PacBio long-reads mapped to GenBank reference NC_007605. Shown in yellow and green are selected ORF and transcripts. Shown in blue are the coverage graphs (solid blue indicates a single, the longest, contig aligned). Individual reads are green (forward) or red (reverse). Reads in yellow are non-unique mappings.

DNA from the BCBL-1, JSC-1, VG-1, BC3, and BC-1 cell lines was subjected to high-fidelity (PacBio HiFi), long-read sequencing. This approach yielded complete, repeat-corrected KSHV^BCBL-1^ (OR117738), KSHV^BC3^ (PQ787490), KSHV^VG-1^ (PQ899224), KSHV^JSC-1^ (PQ868890), and KSHV^BC-1^ (PQ868889) genomes (Fig. 2A through C) and the EBV ^BC-1^ and EBV ^JSC-1^ genomes (Fig. 2D through F). For this experiment, the viruses were not reactivated, and viral DNA was not enriched; instead, total high molecular weight DNA was used, assuming that the KSHV plasmids were present in multiple copies per cell (60–63). In the circular plasmids, the TRs are continuous and linked to unique sequences on either end. Average coverage was 20× for the human genome and higher for the multi-copy KSHV plasmids (Fig. 2F). We compared two approaches to genome assembly. First, we mapped reads directly to the reference genome. Coverage by long-read NGS was lower than by short-read NGS, but PacBio HiFi sequencing approximates Q50 base-pair quality, which exceeded that of Sanger sequencing (Q20). Second, we *de novo* assembled the viral genomes from human-sequence-depleted reads. Long-read NGS yielded a single contig. *De novo* assembly more accurately captures insertions or sequences not previously reported. There were none.

### Additional full-length viral genomes from KSHV endemic regions

Phylogenetic and structural predictions are only possible due to publicly shared data. We and others continue to determine and share high-quality, full-length KSHV genomes through GenBank. Due to human subject protection regulations, many entries contain only minimal identifying information. Here, we report additional pre-cancer, pre-cART treatment KSHV genomes from HIV-positive participants (31) using targeted amplification (Table 1). All primer sequences were removed before mapping. The average coverage for the KSHV genomes isolated from FFPE biopsies was 12,206-fold. The coverage for KSHV genomes derived from KS biopsies was significantly higher than the average genome coverage isolated from plasma, which was 199-fold. This difference was consistent with KSHV biology. In the absence of concurrent KAD, the KSHV DNA copy number in the plasma of HIV-positive KS patients is low compared to other herpesvirus infections (6, 31, 32, 64, 65). Compared to plasma, DNA copy number is higher in the saliva (47, 66–68). For benchmarking, note that the genome quality of early GenBank entries, and presumably the KSHV reference genome, had a minimum coverage of 12-fold (22).

Calling KSHV genomes full-length based on short-read NGS alone is a misnomer because short reads cannot resolve longer repeat regions. These repeat regions include, most notoriously, the terminal (TR) and LANA central domain repeats (69). These pose problems in the initial targeted enrichment, sequencing, and alignment. For clinical samples, separate PCR amplification followed by Sanger sequencing of these regions was conducted (32). Because hypervariable regions are also hyper-recombinogenic, two different batches of the same cell line may have differing numbers of these repeats. The GenBank entry represents the most prevalent variant for repeat regions (TR, ori-L, ori-R, LANA, K15). One way to overcome the conundrum of herpesvirus repeats was to deploy long-read sequencing.

### Phylogenetic reconstruction of KSHV circulating in KS endemic regions

To reconstruct the evolution and population structure of KSHV lineages circulating today, *n* = 164 full-length genomes isolated from Africa were used to construct the Bayesian ML phylogenetic tree. The tree was constructed using an alignment of the central conserved LUR of KSHV (from ORF16 to ORF58) between the two Ori-Lyt sites comprising 65,840 bp of continuous sequence. The LUR excluded the hypervariable K1 and K15 genes, which are subject to strong selection and less helpful in discerning long-term evolutionary patterns. Put differently, nucleotide substitutions within the LUR likely represent genetic drift driven by random single nucleotide mutations. In contrast, SNVs outside the LUR are closely linked to K1 and K15 genotypes. These two genes are under heavy immune selection. This selection may include convergent evolution and recombination, which pose difficulties for phylogenetic topology reconstruction.

Building a phylogeny on just the LUR tests the hypothesis that there is continuing drift of KSHV in endemic regions, where the virus is primarily transmitted from mother to child during the early years of infancy (8, 70–72). The transmission pattern in SSA differs from the MSM cohort of PLWH in the US, which may have started with a recent, single seeding event (73). As a result of different patterns of evolution, functional differences in infectivity, reactivation from latency, replication, and transmissibility may have evolved differently and now define KS in SSA.

**Fig 3:**
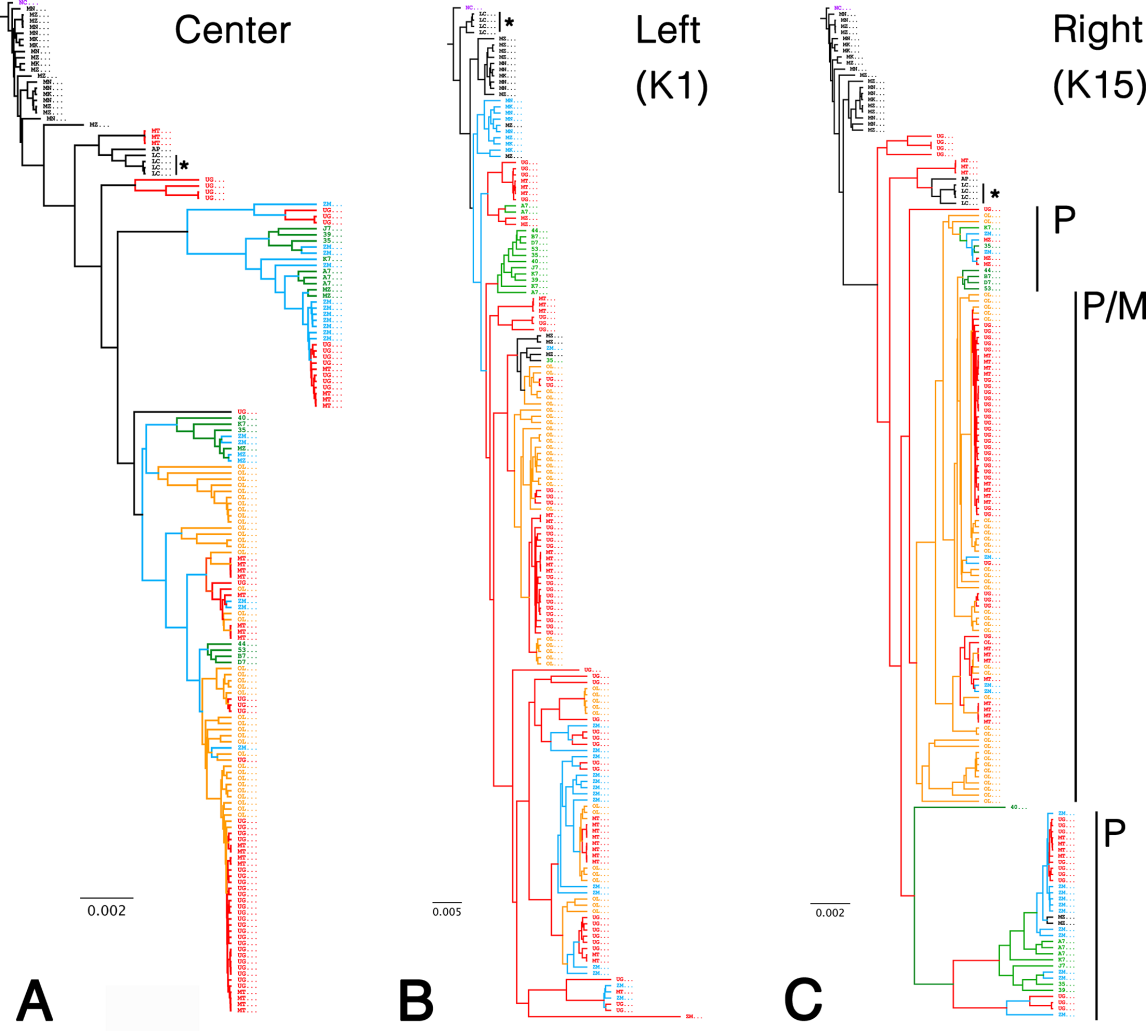
Phylogenetic analysis of LUR, K1, and K15. The results of a Bayesian phylogenetic analysis of the African KSHV genomes using BEAST are shown. (**A**) A tree based on the LUR extracted from 164 genomes, the ML tree identifies two subtypes of KSHV genomes isolated from Africa. The country of origin is indicated by color: Malawi in green, Uganda in red, Zambia in blue, Cameroon in yellow, and USA/Europe/Japan in black. The four Japanese isolates are indicated by an asterisk. (**B**) A tree based on the K1 region (right side). The additional Malawian genomes described in this study are classified as the A5 subtype. (**C**) A tree based on the K15 gene (left side). The additional Malawian genomes described in this study are classified as the P-subtype.

When the tree was rooted in the European KSHV reference genome NC_009333 (GK18), the topology confirmed the existence of the two subtypes of KSHV within SSA (32) (Fig. 3A). All European or USA genomes clustered together and apart from the SSA genomes. Each SSA subtype contains genomes from the East African countries of Zambia (45), Uganda (46, 47), and Malawi (32), suggesting a concurrent circulation of both subtypes. Since the different studies used different sequencing technologies, the two subtypes are not the result of method biases. Genomes isolated from the West African Nation of Cameroon (48) fell within one sublineage. Except for some genomes from Uganda, the SSA KSHV genomes did not intermix with the European ones. The KSHV genomes reported from Japan clustered apart from European or African isolates (Fig. 3, asterisk) (74). The genomes obtained in Malawi, as described in this study and previously, were characterized as either lineage A or B based on the tree of the LUR. We identified 20 non-synonymous variants in sublineage A and five in sublineage B. An additional six non-synonymous variants were shared across all lineages (Table 2).

To extend our analysis beyond the central region, we explored alignments of regions to the left and right of the central LUR. The left side harbors the hypervariable K1 gene and the left origin of replication (Fig. 3B). The hypervariable region within the K1 ORF drove the phylogeny, as genomes clustered by their K1 subtype. The new genomes reported here were of the A5 subtype, typical for African isolates (75–77) and separated from the European and typically older strains of the C3 subtype. The phylogeny to the right of the LUR was driven by the hypervariable K15 gene (Fig. 3C), as genomes clustered by their K15 subtype (30, 78, 79). The new genomes described in this study are of the P subtype. Based on K15 and adjacent regions of the viral genome, the African isolates clustered apart from the early US and European isolates.

**Fig 4:**
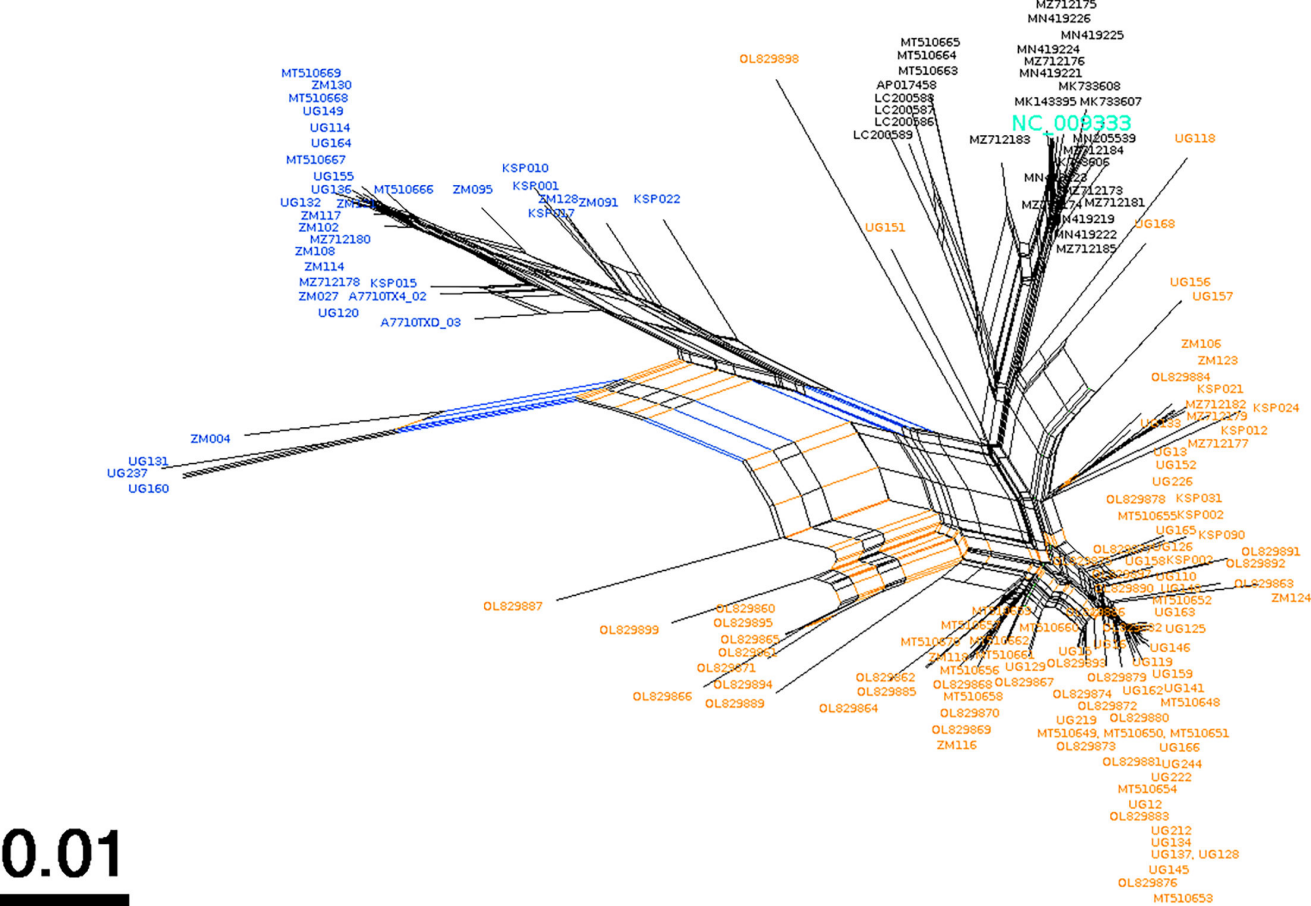
SplitTree analysis. This analysis used *n* = 164 KSHV LUR sequences to construct the ML phylogenetic tree. Darker branch shadings represent branches with higher confidence (1,000 bootstrap replicates). The Phi test for recombination found statistical evidence for recombination (*P* < 0.01). A total of 1,393 informative sites were found using a window size of 100. Mean = 0.3132622742; variance = 4.70900; observed = 0.093065.

A SplitTree network analysis (80) on the LUR was conducted to formally investigate recombination events. In contrast to the ML trees above, this analysis was unrooted. The SplitTree network algorithm also divided the genomes into three partitions consistent with the rooted ML tree. The darker shading of the branches in the SplitTree network corresponds to the higher confidence of the branch. The SplitTree analysis supports the phylogenetic clustering of the genomes into three sublineages, two consisting of recent genomes from SSA and one containing the genomes from Western Europe and the US (Fig. 4). Within this network, a Phi test indicated statistically significant evidence for recombination (*P* < 0.01). These findings substantiate the genetic variability found within the LUR region of KSHV and provide evidence for recombination events within the evolutionary history of KSHV.

In conclusion, KSHV seems to drift over time and space, with more recent genomes from KSHV endemic regions separating into two lineages and differing from calendar-date-older sequences collected from US and European isolates. This conclusion, however, is confounded by the fact that sequencing technologies and bioinformatics assembly methods also developed over calendar time.

### AlphaFold predictions of proteins with shared African SNVs

Next, we modeled the structure of proteins that carried non-synonymous, shared SNVs in our phylogenetic analysis using AlphaFold2. We focused on ORFs with SNVs that resulted in an amino acid change with different chemical characteristics as these more likely result in conformational changes.

### ORF49

**Fig 5:**
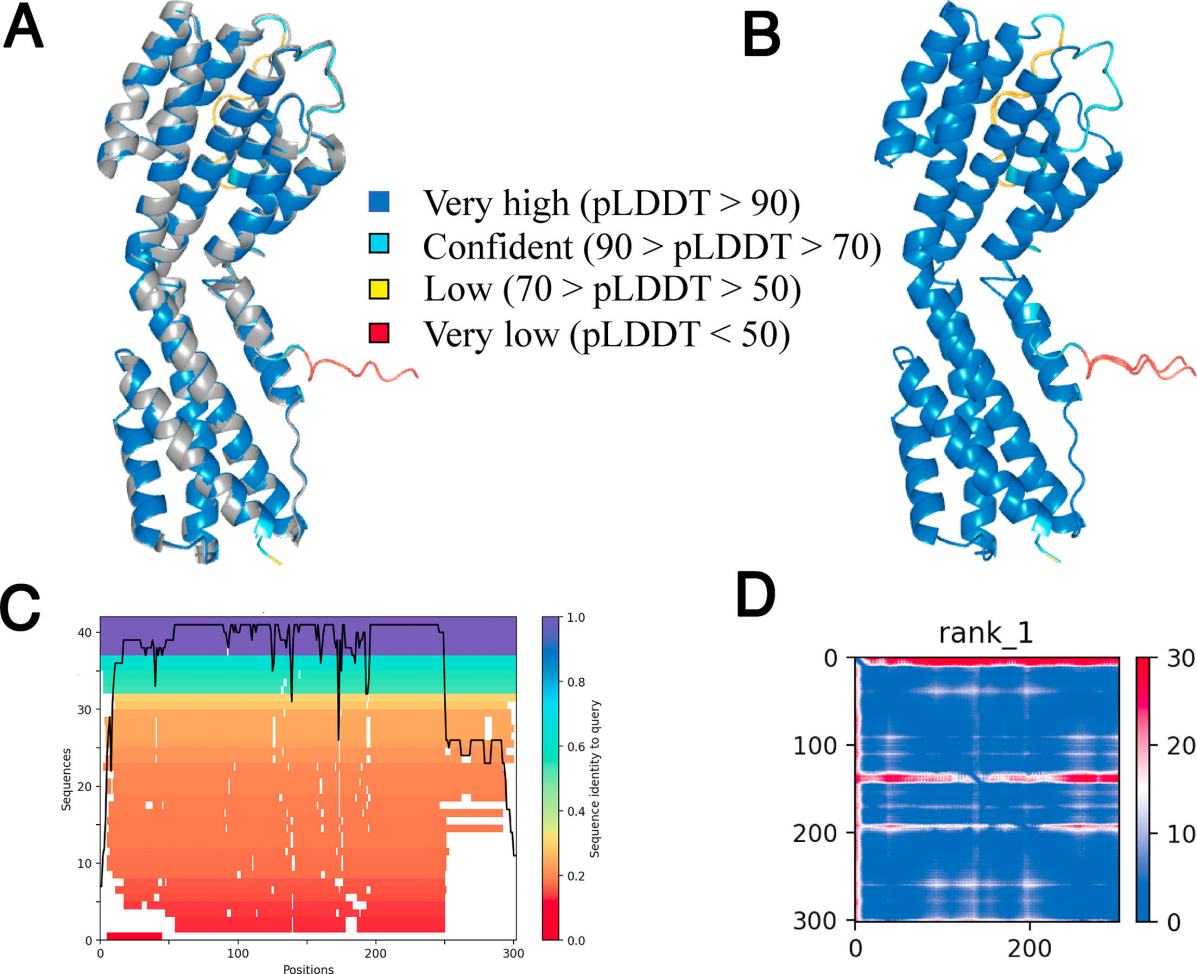
Model of KSHV ORF49. (**A**) The ORF49 crystal structure (5IPX.pdb) in gray is aligned to the AlphaFold2 model for the reference sequence, as shown in the pLDDT color scoring. (**B**) The highly confident model of the ORF49 sequence from the Malawian isolates is colored in the pLDDT color scoring. (**C**) The sequence coverage plot indicates that over 40 sequences were used to generate the MSA and their sequence similarity to the query ORF49. The sequences were 80%–100% similar to ORF49, except at the N- and C-terminals. (**D**) PAE indicates that AlphaFold2 is highly confident that the predicted structure has two domains and spatial arrangement. The axes represent the residue index, i.e., position of the amino acid.

**Fig 6:**
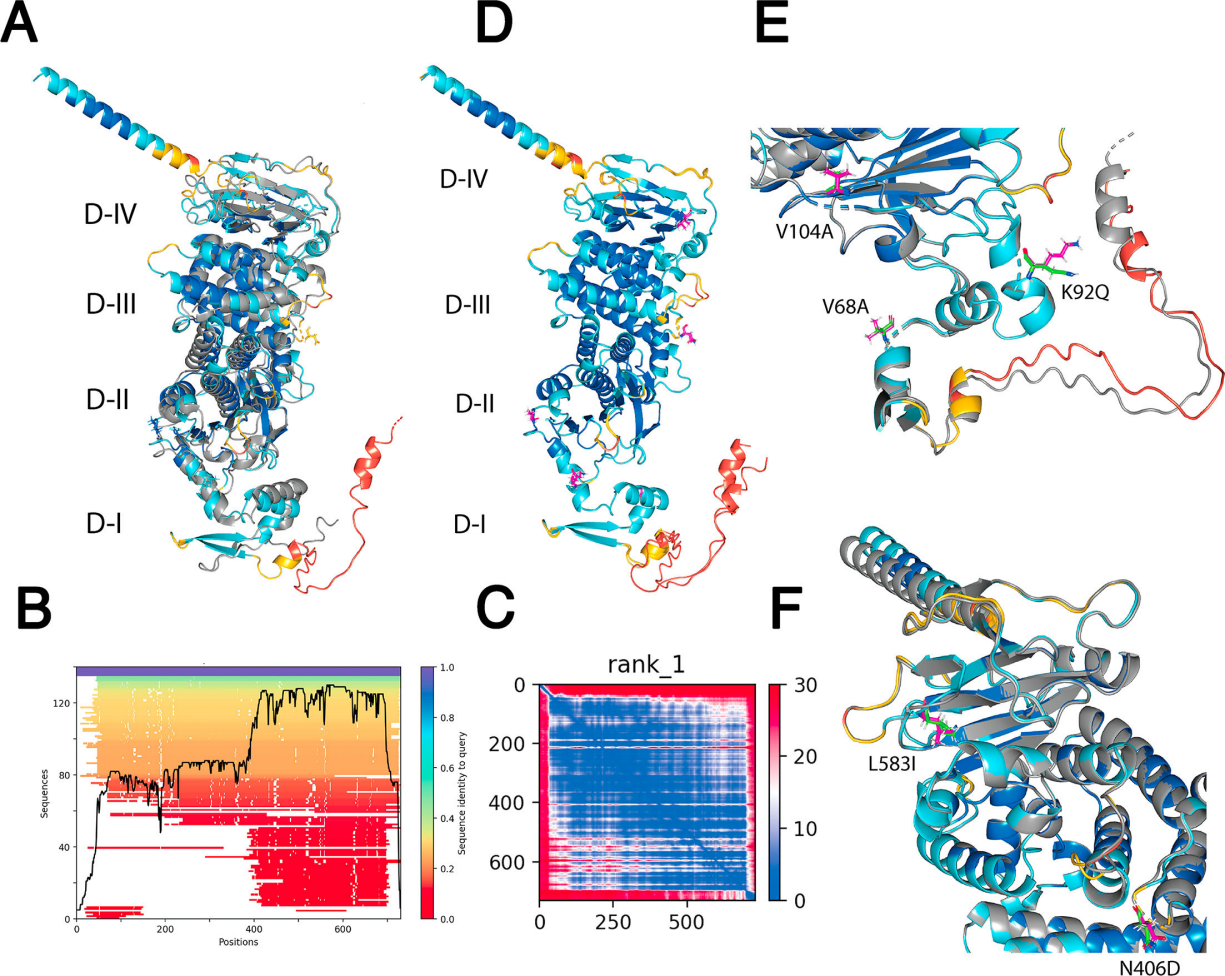
AlphaFold2 model of KSHV ORF22/gH. (**A**) The ORF22 crystal structure (7czf.pdb) in gray is aligned with the reference model, as shown in the pLDDT color scoring. The domains of the structure are indicated. (**B**) The sequence coverage plot indicates that 140 sequences were used to generate the MSA and their sequence similarity to the query ORF22. The sequences were 40%–60% similar to ORF22, except at the N- and C-terminals, where similarity was 20%. (**C**) PAE indicates that AF2 is highly confident the predicted structure is multidomain and in its spatial arrangement. The axes represent the residue index, i.e., position of the amino acid. (**D**) The highly confident reference model of ORF22 aligned with the model of MZ712180 of subtype A and was colored in the pLDDT color scoring. The structures are almost identical except for the N- and C-terminal misalignment and the additional alpha helix at the N-terminal. (**E**) The two non-synonymous SNVs identified in the D-I domain are V68A and V104A. (**F**) The two non-synonymous SNVs are found in the D-III and D-IV domains, N406 and L583I, respectively (green line residue represents the reference sequence, and pink stick residue represents the Malawian sequence).

The AlphaFold2 model of ORF49 was similar to the crystal structure, color coded in gray (RMSD = 0.273 Å) (Fig. 5A). The ORF49 structure consists of 12 alpha-helices bundled into two pseudo domains. In Fig. 5 and 6; Fig. S1A always shows the model with the pLDDT score is color coded, such that cooler colors (blue) indicate higher prediction confidence. The pLDDT confidence scores of the reference sequence indicate a very high confidence model as indicated by the blue color. Variability is noted in three places: first, at the N-terminal loop (red color in Fig. 5A) where the crystal structure is missing nine residues; second in the loop region on top of the structure (cyan color in Fig. 5A), which is disordered in the crystal structure; third for a stretched helix in the back (cyan color in Fig. 5A). The predicted AlphaFold model of the Malawian isolate, MZ712182 (subtype A), was identical (RMSD = 0.038 Å) (Fig. 5B) as ORF49 is highly conserved.

The sequence coverage plot indicates that AlphaFold2 used 40 sequences with a high sequence similarity across the central region of the protein, amino acids 50 to 250 and less similarity at the N and C terminus (Fig. 5C). The PAE plot (Fig. 5D) indicated a low PAE at residue pairs x and y, translating to a well-defined model of two domains (in blue) separated by a flexible region around amino acid 150 in the middle (33). The N-terminus also has low (red) PAE scores. The ORF49 protein reconstruction thus serves as the positive control and benchmark for subsequent predictions, as it has a ground truth in the crystal structure of the reference sequence.

### ORF22/gH

The AlphaFold2 model of gH aligned to the crystal structure (extracted from the PDB file and removing gL and the LBD) with an RMSD = 1.875 Å (544 atoms) (Fig. 6A). The model for gB was 10-fold less accurate than the model for ORF49 if measured against a single crystal structure. Some of the secondary elements are not superimposed. Several reasons may have contributed to this. Firstly, the gH crystal structure lacks 35 and 32 residues at the N-terminus and C-terminus, respectively. These residues comprise an extended loop region in the AlphaFold2 model, with predicted alpha helices and beta sheets, i.e., not unordered. Secondly, within the regions of high confidence, the misalignment of the alpha helices in the D-II domain could contribute to the high RMSD, as well. AlphaFold2 predicted an additional small alpha helix in the D-II and two small alpha helices in the D-III domain of a putative gH monomer absent in the crystal structure of gH: gL: EphA2 trimer. The model used 140 sequences to generate the MSA (Fig. 6B), with the highest conservation in the region between amino acids 400 to the end, i.e., the C-terminal half of the protein had twice as much data as the N-terminal half. Nevertheless, the PAE indicates high confidence in the overall protein orientation (Fig. 6C).

Next, we compared the AlphaFold2 models of the reference sequence and the Malawian isolate from subtype A, MZ712182, and B. The subtype A aligned to the reference model with an RMSD = 0.137 Å (557 atoms) (Fig. 6D) and to the genome from subtype B with an RMSD = 0.201 Å (588 atoms). The model for subtype A aligned to the one of subtype B with an RMSD = 0.074 Å (570 atoms), indicating similar folding except for an additional alpha helix observed at the N-terminal and a misalignment in the loop region of D-IV. The reference sequence and Malawian isolate models aligned with the same secondary structures, except the extended helix in the D-I domain, which is shortened in subtype B and not present in subtype A. The misaligned regions include the extended loop in the D-I domain and the loop in the D-III domain (Fig. 6D).

These structural differences may be due to the four non-synonymous SNVs found in most African genomes and not in the reference strain (Fig. 6E). Two of the four SNVs are in the D-I domain. They include Val68Ala (86% frequency in subtype A; 99% frequency in subtype B) and Val104Ala (86% in subtype A; 99% in subtype B). The valine to alanine change at positions 68 and 104 are small hydrophobic amino acids, with the latter on a loop structure connecting two alpha helices. Val68Ala is on the end of an N-terminal alpha-helix. The N-terminus of ORF22 interacts with gL and the human EphA2 (39). The Val68 forms two contacts with the Leu66 residue on the alpha helix (2.2 Å and 3.0 Å). Those are likely preserved in the Ala68 variant. The Val104 forms contacts with residues on the beta-sheet; the carbonyl group of Val104 forms a hydrogen bond with the residue Ala244 (2.0 Å), and the amino group of Val104 forms a hydrogen bond with Phe243 (1.9 Å). The carbonyl and amino group of Ala104 in the Malawian genome form the same hydrogen bonds as Val104, with a slightly shorter distance of 1.8 Å between the amino group and Phe243 compared to the reference genome, and the carbonyl group of Ala104 forms a hydrogen bond with Ala244 (2.0 Å).

The two non-synonymous SNVs found in the C-terminal include the Asn406Asp (87% in subtype A; 100% in subtype B) in the D-III domain and Leu583Ile (87% in subtype A; 100% in subtype B) in the D-IV domain (Fig. 6F). Leu583 in the reference genome forms a hydrogen bond with its amino group to Ser603 (2.8 Å) and the carbonyl group to Ala586 (2.6 Å). The Ile583 variant in the African subtypes likely maintains those interactions. These findings have immediate value, considering that these SNVs represent replication-competent, common KSHV variants. They could become hotspots for escape mutants to antibodies targeting KSHV gH or engineering targets to increase the stability of gH and thus the infectivity of KSHV bacmids.

### ORF34

There is no crystal structure of ORF34. Thus, the basis for AlphaFold2 prediction is weaker than the preceding targets (Fig. S1). The models based on the Malawian isolates differed from the reference genome and from each other. The highly conserved C-terminus of the protein aligned to subtype A, MZ712180, with an RMSD = 0.248 Å (208 atoms), and to subtype B, MZ712182, with an RMSD = 0.156 Å (220 atoms). The N-terminus differed between the African subtypes. The C-terminus of the protein aligned perfectly across all models, consistent with its conserved function and interactions. This study identified four common, non-synonymous variants in ORF34. The Ala225Ser (82% in subtype A; 98% in subtype B) was present in almost all African isolates. Leu64Gln (81% frequency) and Glu96Lys (85% frequency) are located in the N-terminus and are only present in subtype A and Cys61Ser (94% frequency) in subtype B. These were located on loop regions of the protein and modeled with low confidence. Cys61Ser in subtype B may be responsible for the loss of the terminal alpha helix, which is present in both subtype A and the reference genome. If true, this represents a drastic change in the protein structure. The conserved amino acids in the N-terminal of ORF34 remain conserved in all genomes described here.

Exploring every non-synonymous SNV in every one of the 80 proteins of KSHV by AlphaFold2 is not feasible. Also, the sequence space of KSHV variants is still limited, as is structure prediction accuracy. Nevertheless, these examples provide a blueprint for high-throughput evolution to structure experiments.

## DISCUSSION

This study added 13 complete KSHV genomes to the growing collection of publicly available KSHV genomes isolated from KS endemic areas in recent calendar time (6, 22, 32, 45–48, 64, 65, 81, 82). It extends community efforts to assemble a comprehensive collection of complete viral genomes representing KSHV diversity today. The samples were collected from HIV-positive adult KS patients at study enrollment, i.e., prior to any KS treatment (31). Eight samples were from plasma, and seven were from KS tumor tissue. One pair of samples was matched, i.e., obtained from different compartments for the same participant.

Furthermore, this study added KSHV^BC-1^, KSHV^BCBL-1^, KSHV^BC-3^, and KSHV^JSC-1^ genomes as ascertained by long-read sequencing of latent virus and *de novo* assembly. The initial genomes were remarkably error-free already, given that they were assembled by Sanger Sequencing at ~12× coverage (22). We also obtained the complete human genomes for these cell lines, extending our earlier studies (83).

Even though the study isolated DNA from 137 plasma samples and 75 KS FFPE blocks, PCR-based targeted enrichment and sequencing succeeded only for 6% of the plasma and 8% of the FFPE blocks. Success was defined as a sequencing depth sufficient to identify SNVs with high confidence across the entire genome. Several explanations exist for the limited yield of complete genomes from “field samples.”

First, concerning sequencing from FFPE blocks. Preserving samples collected under routine clinical practice conditions in low- and middle-income countries will always be sub-optimal. Improper (wrong pH) storage or over-fixation (due to lack of personnel) of tumor biopsies is associated with DNA degradation and fragmentation. While DNA fragmentation is less a problem for diagnostic PCR and limited sequencing of individual genes, it dramatically diminishes the quality of whole genome assembly, which at its core assumes even and equal coverage across the targeted genome.

Second, concerning sequencing from plasma. The DNA copy numbers for KSHV are an order of magnitude lower than saliva or lesion-associated genome copy numbers (66, 84–86). The KSHV plasma DNA copy number is typically lower than the EBV or HCMV DNA copy numbers in affected individuals, rarely rising above five log_10_ copies/mL. Extraordinarily high plasma genome copy numbers usually indicate the presence of concurrent KAD, such as MCD or KICS (64, 65). This difference in DNA copy number may explain why GenBank has more complete EBV (*n* ~1,375) than KSHV (~255) genomes. A higher level of performance is required to attain complete KSHV genomes from KS patient material than from EBV or HCMV carriers.

The KSHV genomes used for phylogenetic analyses were from GenBank. Acceptance in GenBank assures a certain level of quality control but only minimal association with metadata. The entries or derivatives are publicly available and indefinitely preserved. The GenBank identifiers are unique, and the coding regions are annotated to match the KSHV reference sequence. Additional sequence information for KSHV exists as unassembled reads in SRA archives, e.g., bio project PRJEB2768. Some of this information was used to assemble some KSHV genomes for phylogenetic analysis; however, since we did not do the sequencing, it remains for the authors to submit those sequences to GenBank.

The phylogenetic analysis presented here confirmed our prior discovery of two distinct KSHV lineages co-circulating in KSHV endemic countries today (32). This updated analysis gained greater power and granularity by adding data generously shared by others (6, 22, 32, 45–48, 64, 65, 81). The extended phylogenetic analysis uncovered several non-synonymous SNVs common to each clade or prevalent within particular countries. The extended phylogenetic analyses separate current African KSHV isolates from earlier European and US isolates.

The four Japanese KSHV strains clustered separately. These isolates are from classic KS cases and are classified as the C subtype of K1 and M based on their K15 allele. The virus sequences have approximately 500 non-synonymous mutations compared to the reference strain. Most of those are shared among them; however, there is a distinction between the three genomes from the Miyako islands and the one from mainland Japan. In an easily overlooked publication, Awazawa et al. reported that these genomes were collected from classic KS patients living on the Miyako Islands in Okinawa Prefecture at the Southernmost tip of Japan (74). On the Miyako Islands, KSHV seroprevalence is 15.4%, which is significantly above mainland Japan, the US, or Northern Europe. The separation in sequence space for the Japanese KSHV strains supports our contention of continuous KSHV sequence drift, here uncovered by separation in geographical space.

Geographic isolation has been one of the fundamental mechanisms of speciation ever since Darwin documented different finch species on different islands within the Galápagos Archipelago (reviewed in reference [87]). It applies to KSHV evolution as well. The herpesviruses evolved with human migration as early as the Bronze Age (88, 89). Then, geography separated the KSHV strains circulating in areas of moderate to high prevalence (15%–75%) in Japan, Africa, Europe (mostly the Levant), and the Uygur populations in Xinjiang, China (90). A limitation of this conjecture is that we only have enough sequence isolates from SSA to exclude sample bias and founder effects.

For the high-coverage genomes, low-frequency SNVs could be identified with high confidence. This, together with the formal detection of recombination events, supports the notion of reinfection, intra-host evolution, or concurrent reactivation events within a lesion, a person, or a saliva sample (46). How frequent these are and whether they are linked to particular environmental, clinical conditions, or age is subject to further study. None of the recombinant KSHV genome sequences in a mixed infection lesion have been evaluated with regard to replication competency. Hence, there exists the possibility that these represent defective rearrangements.

The severe paucity of high-resolution structures for most KSHV proteins remains a barrier to inferring biological consequences of sequence variation. Machine learning programs have recently pushed the boundaries of protein structure prediction. These programs provided models to put some viral SNVs into a structural context. AlphaFold2 was used here due to its superiority over other algorithms in determining protein structures (33). There are limitations specific to each protein, however. Each prediction has to be considered based on the underlying evidence to build the model. Some KSHV ORFs are homologous to other herpesvirus ORFs, suggesting a conserved fold. For some viral ORFs (KSHV and other herpesviruses), CryoEM or X-ray structures were incorporated by AlphaFold2. For other viral ORFs, there is little to no information beyond general folding tendencies to derive a structure. We consider such modeling approaches as hypothesis-generating, which has value in itself. Future experimental verification of KSHV protein structures is urgently needed. It is unclear how adding additional, more diverse KSHV protein sequences will affect structure prediction. Will AlphaFold get worse, begin to “hallucinate” even, and how would we find out? We opine that obtaining more high-resolution structures of KSHV protein variants, i.e., ground truth data, is as important as obtaining more genomic sequences.

Several SNVs mapped to KSHV surface proteins and regulatory proteins. Mapping SNVs to the model for gH may suggest stabilizing mutations for crystallography, and it may inform vaccine design. By severely straining credulity, one could argue that some SSA-specific and conserved SNVs represent receptor usage patterns based on ephrin alleles prevalent in endemic areas but not in US and European populations (91, 92). This would explain the non-universal distribution of KSHV, which is unusual among human herpesviruses.

## SUPPLEMENTAL MATERIAL

Figure S1 jvi.01950-24-s0001.docx AlphaFold2 model of KSHV ORF34.

ASM does not own the copyrights to Supplemental Material that may be linked to, or accessed through, an article. The authors have granted ASM a non-exclusive, world-wide license to publish the Supplemental Material files. Please contact the corresponding author directly for reuse.
