# Supplementary material for: Genome evolution of Kaposi sarcoma-associated herpesvirus (KSHV)
Source: J Virol. 2025 Apr 16;99(5):e01950-24. doi: 10.1128/jvi.01950-24 (PMC12090769; doi:10.1128/jvi.01950-24)
Supplement: Figure S1 — AlphaFold2 model of KSHV ORF34. [file jvi.01950-24-s0001.docx]

**Supplemental Figure 1:**

**AlphaFold2 model of KSHV ORF34. (A)**The predicted reference sequence model of ORF34 is shown in the pLDDT color scoring. **(B)**The reference sequence model is colored in grey and aligned to the genomes from subtypes A and B, which are colored in the pLDDT scoring. The genomes align perfectly at the C-terminal but are unaligned at the N-terminal. **(C)** The sequence coverage plot indicates that over 100 sequences were used to generate the MSA and their sequence similarity to the query ORF34. The sequences were 60-100% similar to ORF34, except at the C-terminal, where similarity was 20-40%. **(D)**Predicted Aligned Error (PAE) indicates that AF2 is highly confident the predicted structure has two domains and is less confident in their spatial arrangement relative to one another. **(E)**The two nonsynonymous SNVs are identified in the genomes of subtype A, L64Q, and E96K. (**F)** The nonsynonymous SNV was found in the genomes of subtype B, C61S (green line residue represents the reference sequence, and pink stick residue represents the Malawian sequence).

Approximately 100 genomes were included in generating the MSA, with high sequence identity across (60-100%) except for the C-terminus. The PAE plot shows that there are two domains of IRF34 but that the spatial arrangement of the domains relative to one another has a low confidence. The confidence of the N-terminus is variable, and the loop regions are modeled with very low confidence. The alpha helices and beta sheets have a low to medium confidence score.

**
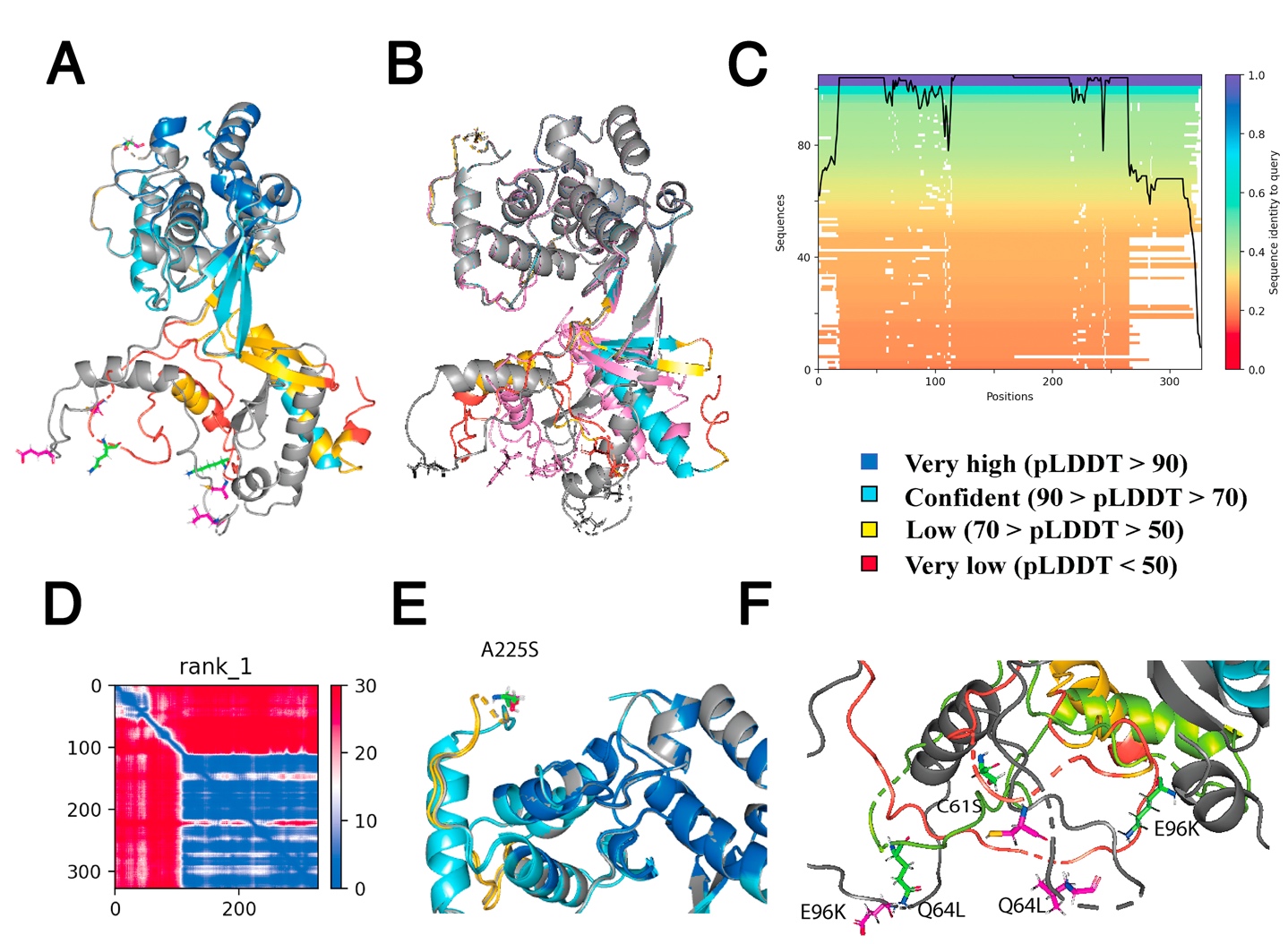
**
